# Treatment of hypertension during pregnancy: a cohort of pregnancy episodes from the SIDIAP database, Catalonia, Spain

**DOI:** 10.3389/fphar.2024.1346357

**Published:** 2024-06-17

**Authors:** Ainhoa Gomez-Lumbreras, Carles Vilaplana-Carnerero, Marta Lestón Vázquez, Cristina Vedia, Rosa Morros, Maria Giner-Soriano

**Affiliations:** ^1^ Department of Pharmacotherapy, College of Pharmacy, University of Utah, SLC, UT, United States; ^2^ Fundació Institut Universitari per a la Recerca a l’Atenció Primària de Salut Jordi Gol i Gurina (IDIAPJGol), Barcelona, Spain; ^3^ Universitat Autònoma de Barcelona, Bellaterra (Cerdanyola del Vallès), Spain; ^4^ Plataforma SCReN, UIC IDIAPJGol, Barcelona, Spain; ^5^ Departament de Medicina, Universitat de Barcelona (UB), Barcelona, Spain; ^6^ Àrea del Medicament i Servei de Farmàcia, Gerència d'Atenció Primària Barcelona Ciutat, Institut Català de la Salut, Barcelona, Spain; ^7^ Servei d’Atenció Primaria Maresme, Barcelona, Spain; ^8^ Institut Català de la Salut, Barcelona, Spain

**Keywords:** hypertension, pregnancy-induced, pregnancy outcome, antihypertensive agents, cohort studies, electronic health records, EHR

## Abstract

**Introduction:**

Hypertension during pregnancy is one of the most frequent causes of maternal and fetal morbimortality. Perinatal and maternal death and disability rates have decreased by 30%, but hypertension during pregnancy has increased by approximately 10% in the last 30 years. This research aimed to describe the pharmacological treatment and pregnancy outcomes of pregnancies with hypertension.

**Methods:**

We carried out an observational cohort study from the Information System for the Development of Research in Primary Care (SIDIAP) database. Pregnancy episodes with hypertension (ICD-10 codes for hypertension, I10–I15 and O10–O16) were identified. Antihypertensives were classified according to the ATC WHO classification: β-blocking agents (BBs), calcium channel blockers (CCBs), agents acting on the renin‐angiotensin system (RAS agents), diuretics, and antiadrenergic agents. Exposure was defined for hypertension in pregnancies with ≥2 prescriptions during the pregnancy episode. Descriptive statistics for diagnoses and treatments were calculated.

**Results:**

In total, 4,839 pregnancies with hypertension diagnosis formed the study cohort. There were 1,944 (40.2%) pregnancies exposed to an antihypertensive medication. There were differences in mother’s age, BMI, and alcohol intake between pregnancies exposed to antihypertensive medications and those not exposed. BBs were the most used (n = 1,160 pregnancy episodes; 59.7%), followed by RAS agents (n = 825, 42.4%), and CCBs were the least used (n = 347, 17.8%).

**Discussion:**

Pregnancies involving hypertension were exposed to antihypertensive medications, mostly BBs. We conduct a study focused on RAS agent use during pregnancy and its outcomes in the offspring.

## Introduction

Hypertension disorders during pregnancy complicate between 5% and 10% of pregnancies and are among the frequent causes of feto-maternal morbimortality (Bramham et al., 2014; Williams et al., 2018; Wu et al., 2020). Hypertension during pregnancy has been associated with maternal complications such as stroke or heart failure, and in the fetus, it is associated with intrauterine growth restriction and stillbirth. Globally, hypertension during pregnancy has increased approximately 10% in the last 30 years, though the death and disability rates have decreased up to 30% (Wang et al., 2021). Hypertension can be a preexisting medical condition before the pregnancy (chronic hypertension) or be induced by the pregnancy and diagnosed after 20 weeks of gestation (gestational hypertension) (Williams et al., 2018).

The European Society of Cardiology (ESC) and the European Society of Hypertension (ESH) establish that pharmacology treatment aims to reduce maternal risk while being safe for the fetus (Williams et al., 2018). These guidelines, even with scarce evidence, recommend pharmacological treatment for those women with persistent elevation in blood pressure (BP) (≥150/95 mmHg), with some other guidelines indicating starting treatment for BP ≥ 140/90 mmHg (ACOG, 2019). However, there is no clear threshold for initiating pharmacological treatment for patients with mild hypertension (systolic BP between 140–150 and 160) (Kaimal, 2022). There are five groups of antihypertensive medications: antiadrenergic agents, β-blocking agents (BBs), diuretics, calcium channel blockers (CCBs), and those acting in the renin‐angiotensin system (RAS) agents, including angiotensin-converting enzyme inhibitors (ACEis) and angiotensin receptor blockers (ARBs). Women already undergoing treatment for preexisting hypertension might continue with their antihypertensive medication; however, agents acting on the renin-angiotensin system (RAS) are contraindicated due to the related adverse fetal and neonatal outcomes, and the indication is to switch the antihypertensives with awareness of the pregnancy (Brown et al., 2018; Braunthal and Brateanu, 2019).

Due to the increase in the number of pregnant women with hypertension and the potential implications of pharmacological treatments in pregnancy outcomes, we describe the antihypertensive medications used in a cohort of pregnancies with hypertension diagnoses.

## Methods

This is an observational cohort study of pregnancies with hypertension diagnoses conducted with data obtained from the Information System for the Development of Research in Primary Care (SIDIAP). The SIDIAP database characteristics have been described elsewhere (Recalde et al., 2022). It contains electronic health records (EHRs) of the Primary Care Centers of the Catalan Health Institute (ICS) in Catalonia, Spain, from 2006 of up to 6 million people and almost 500,000 pregnancy episodes, most of which were followed at the sexual and reproductive healthcare services (ASSIR) of the ICS. The EHRs in ASSIR are used by gynecologists and midwives to register variables related with the sexual and reproductive health of women and follow-up of pregnancies, such as date of the last menstrual period or pregnancy start date (PSD), gestational week, date of delivery or pregnancy end date (PED), and termination outcomes. We identified a cohort of pregnancy episodes (n = 327,865) that occurred during 2011–2020 registered at the ASSIR and those pregnancy diagnoses registered in the primary care EHR through International Classification of Diseases 10th at SIDIAP (ICD-10) (WHO, 2019; Lestón Vázquez et al., 2023).

### Cohort definition

A previous study from SIDIAP identified a total of 327,865 pregnancy episodes occurring during 2011–2020 (Lestón Vázquez et al., 2023). For our cohort, we included those pregnancy episodes with ICD-10 codes for hypertension (I10–I15) and gestational hypertension (O10–O16). For patients with more than one ICD-10 code for hypertension, the first one recorded was selected. Based on the date of the registered hypertension code, the pregnancy episodes were classified as chronic hypertension (codes before the PSD) and gestational hypertension (those registered during the pregnancy episode).

Only completed pregnancy episodes were considered, meaning only those pregnancies starting after the study period start date (1 Jan 2011) and completed by the end of the study period (30 June 2020).

### Antihypertension medication exposure

The antihypertensive medications were grouped and defined by the WHO ATC classification as follows: antiadrenergic agents (C02), diuretics (C03), BB (C07), CCB (C08), and RAS agents (C09) (WHO Collaborating Centre for Drug Statistics Methodology, 2022).

SIDIAP pharmacy invoice data were used to define drug exposure. Invoices of those antihypertensive medications prescribed between the previous month and the PSD up to the month preceding the PED were considered to occur during the pregnancy episode. All prescriptions issued in primary care and ASSIR centers of drugs reimbursed by the Spanish National Health System that are dispensed in a community pharmacy produce a register in the invoice data. Pregnancies with at least two invoices for an antihypertensive medication were considered exposed.

### Variables

The demographic characteristics, MEDEA socioeconomic index (Domínguez-Berjón et al., 2024), body mass index (BMI), smoking status, and alcohol intake were considered from 12 months before PSD up to PED. The number of pregnancies by woman was considered if occurring during the study period (2011–2020), with no distinction made regarding pregnancies with multiple fetuses.

### Statistical analysis

#### Sample size and study power

We did not anticipate any specific number of pregnancies as we used all the pregnancy episodes with a diagnosis of hypertension.

#### Main analysis

We calculated descriptive statistics for pregnancy characteristics and antihypertensive medication exposure [mean and standard deviation (SD), median and interquartile range (IQR), or percentages].

#### Results

From the 327,865 pregnancy episodes identified in SIDIAP, a total of 4,839 (1.5%) pregnancy episodes were included in our study cohort. This cohort was built with pregnancy episodes with hypertension diagnosis during the study period (2011–2020). In Table 1 it can be seen that the cohort had 1,944 (40.2%) pregnancy episodes exposed to an antihypertensive medication. Mothers were older in the exposed group (mean age in years 36.0, SD 5.2) than in the non-exposed group (34.2, SD 5.5). The rate of obesity was higher in the exposed group (35.3% vs. 27.2%). Almost three-quarters (73.3%) of the exposed pregnancies had chronic hypertension (diagnosis registered before the PSD) compared to half of the non-exposed pregnancies (53.6%). There was a higher rate of live-birth pregnancies among pregnancies that were not exposed to drugs (82.7% vs. 77.7%) and, on average, live birth pregnancy duration was 1 week longer in the non-exposed group than in the exposed group (mean 39.0 weeks, IQR 36.0–40 vs. 38.0 weeks, IQR 30.3–39.6, respectively). To see all the baseline characteristics of the pregnancy episodes, please see Table 1.

**TABLE 1:** Baseline characteristics of all the hypertension pregnancy episodes classified according to the antihypertensive medication exposure.

Total cohort (4,839 pregnancy episodes)	Exposed to antihypertensive drugs N = 1,944	Not-exposed to antihypertensive drugs N = 2,895	*p*
Mother age (mean, SD) at PSD	36.0 (5.2)	34.2 (5.5)	<0.001
Obesity (ICD-10 code + BMI≥30)	686 (35.3)	788 (27.2)	<0.001
Mother’s socioeconomic status (MEDEA)^a^			
Rural	337 (17.3)	508 (17.5)	0.004
Urban (U)	177 (9.1)	254 (8.8)	
U1	152 (7.8)	311 (10.7)	
U2	255 (13.1)	399 (13.8)	
U3	271 (13.9)	433 (15.0)	
U4	320 (16.5)	458 (15.8)	
U5	431 (22.2)	530 (18.3)	
Smoking habit^a^	359 (18.5)	548 (18.9)	0.830
Alcohol consumption^a^	180 (9.3)	184 (6.4)	<0.001
CKD (ICD-10 code: N18)	28 (1.4)	15 (0.5)	0.001
Parity number			
1st	1,689 (87.2)	2,432 (84.0)	0.006
2nd	211 (10.9)	383 (13.2)	
≥3rd or more	37 (1.9)	80 (2.8)	
Hypertension			
chronic	1,425 (73.3)	1,553 (53.6)	<0.001
gestational	519 (26.7)	1,342 (46.4)	
Live births	1,510 (77.7)	2,394 (82.7)	<0.001
Average of gestation duration (weeks mean, IQR)	39.0 (36.0-40.0)	38.0 (30.3-39.6)	<0.001
Preterm births (ICD-10th code)	331 (17.0)	356 (12.3)	<0.001

^a^ variable up to 12 months before PSD. SD, standard deviation; PSD, pregnancy start day; ICD-10, International Classification of Diseases 10th version; BMI, body mass index; MEDEA, Mortalidad en áreas pequeñas Españolas y Desigualdades socioEconómicas y Ambientales; REF CKD, chronic kidney disease; IQR, interquartile range.

From the non-exposed pregnancy cohort, there were 529 (18.3%) cases with just one invoice of an antihypertensive medication, which did not meet the criteria for exposure. These were predominantly in the third trimester (n = 301 pregnancies, 56.9%), followed by the first one (n = 194 pregnancies, 36.7%). For these single-invoice pregnancies, BBs were leading the list (n = 301 pregnancies, 56.9%). To see the complete description of cases that were considered not exposed though with one invoice of an antihypertensive, see Supplementary Table S1.

**FIGURE 1:**
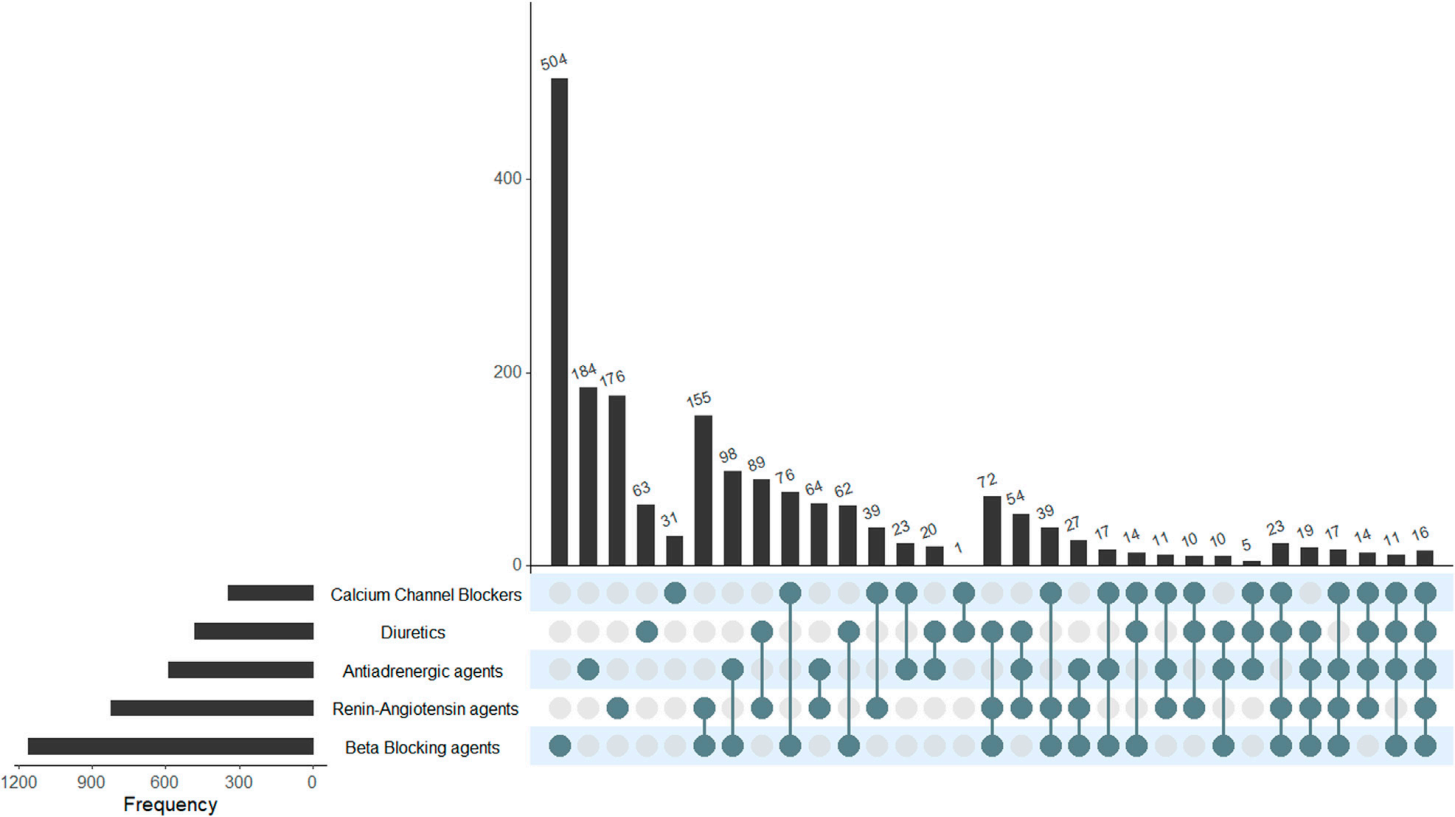
Antihypertensive drugs (and combinations) used across the cohort of exposed pregnancies. This is a two-way reading chart to show the total of unique and combinations of antihypertensive drugs that were used in the pregnancy episodes. Pharmacological groups are shown on the left, with horizontal bars representing the frequency of the pharmacological groups used in monotherapy or in combination. As an example, BB in the horizontal bar shows that they were used in 1,160 episodes, and the longest vertical bar shows the most frequently used treatment, which were BBs alone (n = 504). The most common combination of drugs was RAS + BB, and it is represented in the sixth vertical bar (N = 155).

BBs (n = 1,160 pregnancies, 59.7%) were the most frequently used agents, followed by RAS agents (825 pregnancies, 42.4%). CCBs were the least used (347 pregnancies, 17.8%). The combination of antihypertensive treatments across all the pregnancy trimesters shows BB and RAS agents (155 pregnancy episodes, 8.0%) as the most-used combination, followed by BB agents and antiadrenergic (98 pregnancies, 5.0%). The complete description of the frequency of exposure to the different antihypertensives and combinations through the pregnancies can be seen in Figure 1, and to see the most-used agents for each antihypertensive group, see Supplementary Table S2.

**FIGURE 2:**
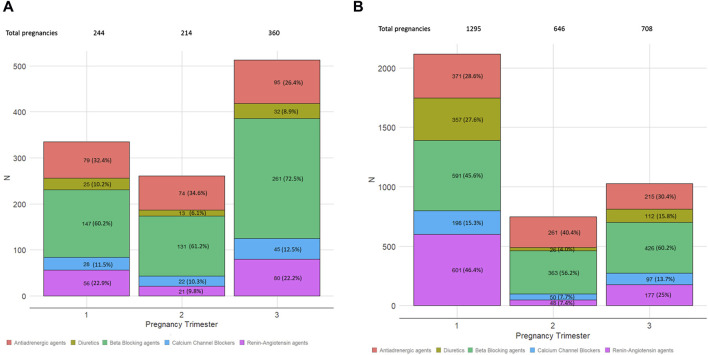
Distribution of the number of pregnancies exposed to the different antihypertensive groups by the trimester of gestation. Each bar represents the three pregnancy trimesters. The five colored boxes represent the pharmacological groups, with the number of episodes and percentage inside each box: antiadrenergic agents, diuretics, beta blocking agents, calcium channel blockers, and renin–angiotensin agents. **(A)** Gestational hypertension exposure. **(B)** Chronic hypertension exposure.

Figure 2A (gestational hypertension) and 2b (chronic hypertension) showed a decrease in exposure during the second trimester. In gestational hypertension (Figure 2A), exposure increased 96.5% from the second to the third trimester, while a 37.3% increase is shown in chronic hypertension pregnancies (Figure 2B). Chronic hypertension pregnancies decreased by 70.5% in exposure to RAS agents from the first trimester to the third (601–177). BBs were the most-used agents across all trimesters for both chronic and gestational hypertension pregnancies.

## Discussion

In this observational cohort study of pregnancy episodes with hypertension in Catalonia, Spain, during 2011–2020, our results showed that more than half of the pregnancies with hypertension diagnosis had no exposure to antihypertensive medications. Among pregnancy episodes with chronic hypertension, almost three-quarters were exposed to antihypertensives, and approximately a quarter of the gestational hypertension episodes were exposed to antihypertensive agents too. The most-used antihypertensives were BBs, and the least-used ones were CCBs. Combinations of antihypertensives were not frequent.

Mothers exposed to antihypertensives were on average 2 years older than those not exposed. Older women are at more risk of hypertension during pregnancy (Khalil et al., 2013). Obesity has previously been associated with hypertension during pregnancy, with up to a three-fold increased risk; accordingly, in our study, the rate of obese women was higher in the exposed pregnancies (Mission et al., 2015). In the literature, these risks have been defined and associated with hypertension during pregnancy (Assis et al., 2008; Poon et al., 2010). Both cohorts, exposed and not exposed, showed a low rate of pregnancy episodes with chronic kidney disease (CKD) (<2%). Among women with childbearing potential, the average rate of CKD is 4%, and our rates seem consistent. We found a higher rate of CKD among those exposed to antihypertensives compared to the non-exposed ones, probably because they have more advanced CKD in need of antihypertensive treatment (Coresh et al., 2007).

Clinical guidelines recommend maintaining pharmacological treatment in women with chronic hypertension when pregnant, except for RAS agents, as they have been associated with adverse perinatal outcomes (Al Khaja et al., 2014; Garovic et al., 2022). Our results showed higher use of antihypertensives in the first trimester among pregnancies with chronic hypertension, in agreement with the guidelines; however, to date, there is no consensus on the BP values to start antihypertensive medication for pregnancies with BP < 160/90 mmHg. Two recently published meta-analyses have shown better outcomes for pregnancies receiving antihypertensive medications, and a network meta-analysis showed that even if all antihypertensives reduce the risk of severe hypertension, labetalol may also decrease proteinuria/preeclampsia and fetal/newborn death (Bone et al., 2022; Attar et al., 2023). The boundaries for BP values for when to start medication are uncertain, making this area suitable for shared decision making (SDM), with some research focusing in developing tools for SDM in women with moderate hypertension (Whybrow et al., 2022).

Our results showed a decrease in the exposure to antihypertensives in the second trimester. During the pregnancy-related physiologic changes, BP usually decreases from the baseline values during the second trimester and increases during the third. These changes in BP may lead to a decision to stop treatment in the second trimester; it is reflected in our results (Sanghavi and Rutherford, 2014).

The reduction in the exposure in chronic hypertension pregnancies by the third trimester might be explained by an early referral of high-risk pregnancies to obstetric departments in hospital settings, with no data in the primary care and ambulatory obstetric settings, as prescriptions from hospital providers were not available. Gestational hypertension pregnancies increased exposure by the third trimester, probably due to the higher BP measures during advanced pregnancy.

The most-used antihypertensive medications during pregnancy were BBs, recommended by obstetric guidelines for non-urgent treatment, where the oral BB labetalol and the antiadrenergic central agent methyldopa are the first-line recommended therapies (Brown et al., 2018; Braunthal and Brateanu, 2019). Our results on the most used group are similar to those of a UK cohort study and a US one, where BBs were the most prescribed agents during pregnancy (Cea Soriano et al., 2014; Garcia et al., 2023). For the second trimester of pregnancy, the UK study showed diuretics as the second most-used group, while in our study, for both chronic and gestational hypertension pregnancies, they were antiadrenergic agents. A French study found CCBs and RAS agents as the second most used after BBs (Lailler et al., 2023). Over a decade ago, another US cohort study described antihypertensive nifedipine (33%), a CCB agent, and methyldopa (26%), an antiadrenergic agent, as the most common drugs (Andrade et al., 2008). A more recent cohort study in North Carolina, US, from 2007 to 2017 showed that BBs (79.2%) were the most used, followed by CCBs (31.8%), with labetalol and nifedipine being the most used agents in these groups (Garcia et al., 2023).

Surprisingly, almost half of the pregnancies were exposed to RAS agents (overall 42.4%), though decreasing by trimester. Our rates of exposure to RAS agents by the third trimester (257, 24%) were higher than those in a UK study (12.5%), but they were much higher than those in a French one (0.7%) (Cea Soriano et al., 2014; Lailler et al., 2023). There is a US cohort study that did not mention any exposure to RAS agents while studying different hypertension disorders (chronic included) (Garcia et al., 2023). RAS should be discontinued as soon as possible with awareness of pregnancy, as continuing exposure through pregnancy has been related to malformations, and this may explain the decrease in their use as pregnancy progresses (Al Khaja et al., 2014; Ahmed et al., 2018).

Several studies have tried to show the association between antihypertension and preterm birth. A meta-analysis of eight randomized controlled trials comparing hypertension treatment to control showed protection of preterm birth (OR 0.69; 95% CI, 0.59–0.82) (Chen et al., 2023). In contrast, the meta-analysis of 16 observational studies found a higher OR (2.23, 95% CI 1.96–2.53) for preterm birth for women with chronic hypertension compared to normotensive, a four times greater odds of medically indicated preterm birth (ORadj 4.76, 95% CI 3.55–6.14) but no association between chronic hypertension and spontaneous preterm birth (ORadj 1.44, 95% CI 0.74–2.80) (Al Khalaf et al., 2021). In 2014, a Cochrane systematic review including 49 trials and over 4,000 pregnant women concluded no effect on the incidence of preterm births of treated mild–moderate hypertension (Abalos et al., 2018).

It remains unclear if treating hypertension resulted in a negative effect on pregnancy outcomes or if the higher risk of preterm birth could be caused by the severity of hypertension. It might be possible that elective delivery could be indicated in those with worse hypertension control and more antihypertensive treatment.

### Limitations

We aimed to describe the use of antihypertensive agents during pregnancy in patients with hypertension disorders considering EHR data potential misclassification in time and specific diagnosis, which was the reason why we classified hypertension as chronic or gestational by the time hypertension diagnosis was registered and not by the specific definition. For hypertension, the BP levels are of relevance, but we did not have BP values, which might have helped in a more accurate classification of hypertension and its severity (Chen et al., 2020). We did not account for multiple pregnancies; these pregnancies have been associated with a higher risk for hypertension and preterm elective delivery (Sibai et al., 2000). To avoid exposure misclassification, we defined hypertension medication exposure by two invoices, considering that just one invoice could be an error, especially when just in the second trimester, or not be accurate for the initial and end terms of the pregnancy. However, exposure misclassification in pharmacoepidemiologic studies conducted with databases has frequently been reported (Prada-Ramallal et al., 2019).

## Conclusion

We have described the antihypertensives used in a Catalan cohort of pregnancy episodes that shows that BBs are prescribed the most, which is in line with worldwide guidelines. Pregnancies were exposed to RAS agents, which deserves further detailed study, as does its implications in the offspring. Considering women already on RAS treatment prior to gestation, physicians may explain the risk of conception while on treatment with these agents.

## Data Availability

The raw data supporting the conclusion of this article will be made available by the authors, without undue reservation.
