# Supplementary material for: Treatment of hypertension during pregnancy: a cohort of pregnancy episodes from the SIDIAP database, Catalonia, Spain
Source: Front Pharmacol. 2024 Jun 17;15:1346357. doi: 10.3389/fphar.2024.1346357 (PMC11215181; doi:10.3389/fphar.2024.1346357)
Supplement: Supplementary file 1 [file Table1.pdf]

## Supplementary Material

**Table S1.** Pregnancies with just one invoice of an antihypertensive medication during the pregnancy by ATC group

| N (%) | Total | Antiadrenergic agents (C02) | Diuretics (C03) | Beta blocking agents (C07) | Calcium Channel Blockers (C08) | Renin-angiotensin system agents (C09) |
|---|---|---|---|---|---|---|
| Total | 529 (100) | 59 (11.1) | 39 (7.4) | 301 (56.9) | 30 (5.7) | 100 (18.9) |
| 1st Trimester | 194 (36.7) | 25 (42.4) | 17 (43.6) | 81 (26.9) | 6 (20) | 65 (65) |
| 2nd Trimester | 34 (6.4) | 6 (10.2) | 2 (5.1) | 21 (7.0) | 3 (10) | 2 (2) |
| 3rd Trimester | 301 (56.9) | 28 (47.4) | 20 (51.3) | 199 (66.1) | 21 (70) | 33 (33) |

**Table S2**. Main active substance exposure by invoice (total invoices 4,022) during the pregnancy episodes and by trimester. Showing those with frequency >1%

| Active Substance | Total Invoices (%) | Invoices Percentage1st trimester (%) | Invoices 2nd trimester (%) | Invoices 3rd trimester (%) |
|---|---|---|---|---|
| Labetalol | 1,022 (25.4) | 604 (20.8) | 475 (45.1) | 658 (39.0) |
| Enalapril | 504 (12.5) | 375 (12.9) | 41 (3.9) | 175 (10.4) |
| Methyldopa | 492 (12.2) | 393 (13.5) | 301 (28.6) | 254 (15.1) |
| Hydrochlorothiazide | 207 (5.2) | 160 (5.5) | 15 (1.4) | 61 (3.6) |
| Beta blocking agents, selective | 200 (5.0) | 170 (5.9) | 18 (1.7) | 41 (2.4) |
| Amlodipine | 183 (4.6) | 149 (5.1) | 31 (2.9) | 50 (3.0) |
| Nifedipine | 143 (3.6) | 55 (1.9) | 34 (3.2) | 83 (4.9) |
| Hydralazine | 109 (2.7) | 59 (2.0) | 36 (3.4) | 59 (3.5) |
| Atenolol | 103 (2.6) | 88 (3.0) | 7 (0.7) | 18 (1.1) |
| Lisinopril | 85 (2.1) | 71 (2.4) | 5 (0.5) | 23 (1.4) |
| Losartan | 78 (1.9) | 73 (2.5) | 10 (1.0) | 12 (0.7) |
| Bisoprolol | 75 (1.9) | 64 (2.2) | 10 (1.0) | 17 (1.0) |
| Ramipril | 69 (1.7) | 55 (1.9) | 7 (0.7) | 19 (1.1) |
